# Correction: Characterization of an intratracheal aerosol challenge model of *Brucella melitensis* in guinea pigs

**DOI:** 10.1371/journal.pone.0218065

**Published:** 2019-06-11

**Authors:** Martha E. Hensel, Daniel G. Garcia-Gonzalez, Sankar P. Chaki, James Samuel, Angela M. Arenas-Gamboa

Fig 3 is incorrectly published as a duplicate of Fig 4. Please view Fig 3 here.

**Fig 3:**
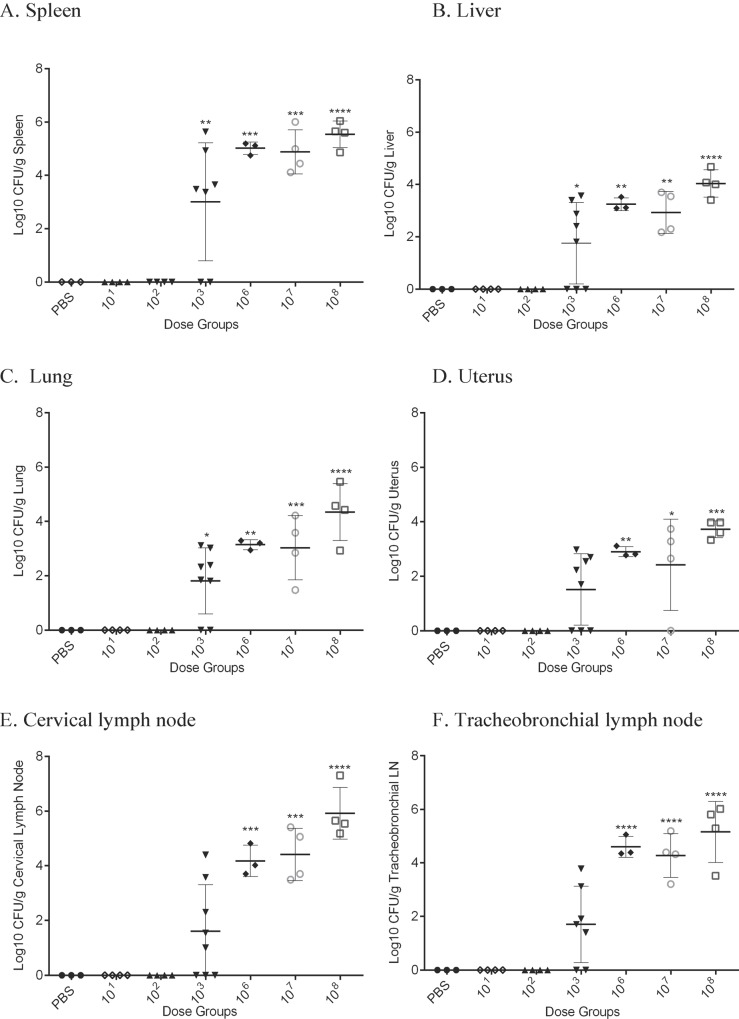
Intratracheal inoculation with *B*. *melitensis* 16M in female Hartley guinea pigs results in systemic infection. Guinea pigs were divided in 7 groups (n = 4) consisting of low dose (10^1^, 10^2^,10^3^), high dose (10^6^, 10^7^, 10^8^), or control (PBS) groups (n = 3). Guinea pigs were inoculated using the MicroSprayer® Aerosolizer and were euthanized 30-days post-inoculation. Colonization was evaluated in the spleen (A), liver (B), lung (C), uterus (D), cervical lymph node (E), and tracheobronchial lymph node (F). The recovery of organisms is plotted as the total CFU/g (means ± standard deviation). Mean recovery per gram of tissue was compared between dose groups and uninfected control guinea pigs. Statistical significance was determined by ANOVA followed by Dunnett’s multiple comparisons. One asterisk, P < 0.05. Two asterisks, *P* <0.01. Three asterisks, *P* <0.001. Four asterisks, *P* <0.0001.
