# Supplementary material for: Characterization of an intratracheal aerosol challenge model of Brucella melitensis in guinea pigs
Source: PLoS One. 2019 Mar 5;14(3):e0212457. doi: 10.1371/journal.pone.0212457 (PMC6400394; doi:10.1371/journal.pone.0212457)
Supplement: S1 Table — Spleen, liver, lung, and uterus were evaluated for type and severity of inflammation, necrosis, and distribution of the lesion from 0 to 4. (PDF) [file pone.0212457.s001.pdf]

**Spleen**	**Score**	**Description**
**Histiocytic inflammation**	0	None
	1	Minimal—One focus per 4x objective
	2	Mild—Two to four foci per 4x objective
	3	Moderate—Five to 10 foci per 4x objective
	4	Marked—>10 per 4x objective
**Neutrophilic accumulation**	0	None
	1	Minimal— few cells identified
	2	Mild—multiple small foci <10 cells
	3	Moderate—1-2 foci of >10 cells
	4	Marked—Multiple foci of >10 cells
**Necrosis**	0	None
	1	Minimal—one focus per 10x objective
	2	Mild—Two to four foci per 10x objective
	3	Moderate—Five to ten foci per 10x objective
	4	Marked— >10 foci per 10x objective

**Liver**	**Score**	**Description**
**Periportal inflammation**	0	None
	1	Minimal— <25% affected
	2	Mild—25%
	3	Moderate—50%-75% affected
	4	Marked—100% affected
**Microgranulomas**	0	None
	1	Minimal—One focus per 10x objective
	2	Mild—Two to four foci per 10x objective
	3	Moderate—Five to 10 foci per 10x objective
	4	Marked—Fills the 10x objective
**Random necrosis**	0	None
	1	Minimal—one focus per 10x objective
	2	Mild—Two to four foci per 10x objective
	3	Moderate—Five to ten foci per 10x objective
	4	Marked— >10 foci per 10x objective

Uterus	Score	Description
**Myometrial inflammation**	0	None
	1	Minimal—One focus per 4x objective
	2	Mild—Two to four foci per 4x objective
	3	Moderate—Five to 10 foci per 4x objective
	4	Marked—>10 per 4x objective
**Endometrial neutrophilic inflammation**	0	None
	1	Minimal— few cells identified
	2	Mild—multiple small foci <10 cells
	3	Moderate—1-2 foci of >10 cells
	4	Marked—Multiple foci of >10 cells
**Edema**	0	None
	1	Present

Lung	Score	Description
**Granulomas**	0	None
	1	Minimal—One focus per 4x objective
	2	Mild—Two to four foci per 4x objective
	3	Moderate—Five to 10 foci per 4x objective
	4	Marked—>10 per 4x objective
**Neutrophilic accumulation**	0	None
	1	Minimal— few cells identified
	2	Mild—multiple small foci <10 cells
	3	Moderate—1-2 foci of >10 cells
	4	Marked—Multiple foci of >10 cells
**Necrosis**	0	None
	1	Minimal—one focus per 10x objective
	2	Mild—Two to four foci per 10x objective
	3	Moderate—Five to ten foci per 10x objective
	4	Marked— >10 foci per 10x objective
**BALT hyperplasia**	0	None
	1	Present
