# Supplementary figures and images for: Characterization of an intratracheal aerosol challenge model of Brucella melitensis in guinea pigs
Source: PLoS One. 2019 Mar 5;14(3):e0212457. doi: 10.1371/journal.pone.0212457 (PMC6400394; doi:10.1371/journal.pone.0212457)

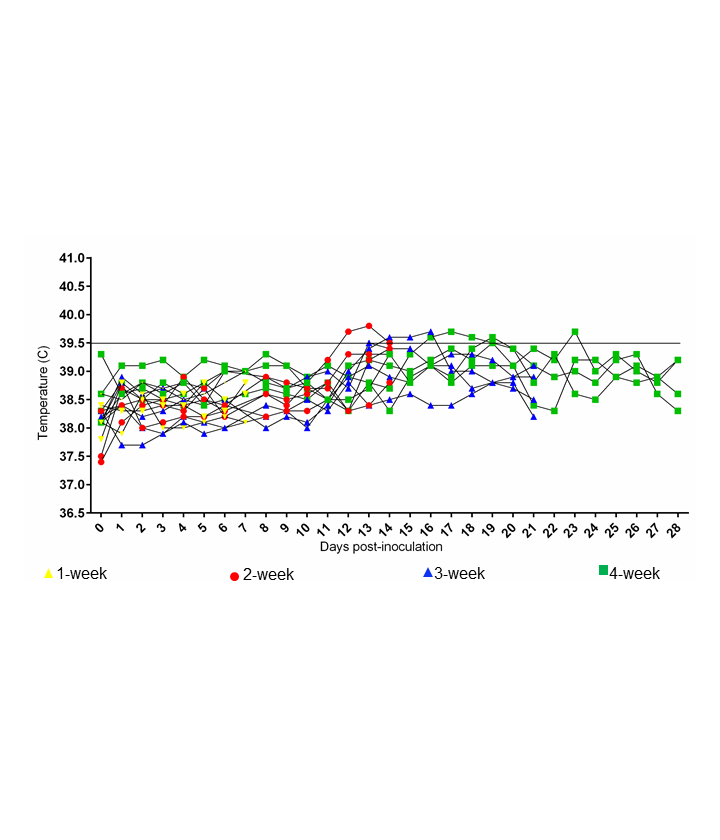

Supplement: S1 Fig — Body temperature changes in guinea pigs (n = 4) after intratracheal inoculation 1x10^7^ CFU B. melitensis 16M. The solid line at 39.5o C indicates the threshold for fever. Guinea pigs developed fever beginning at day 12 post-infection. (TIF) [file pone.0212457.s002.tif]

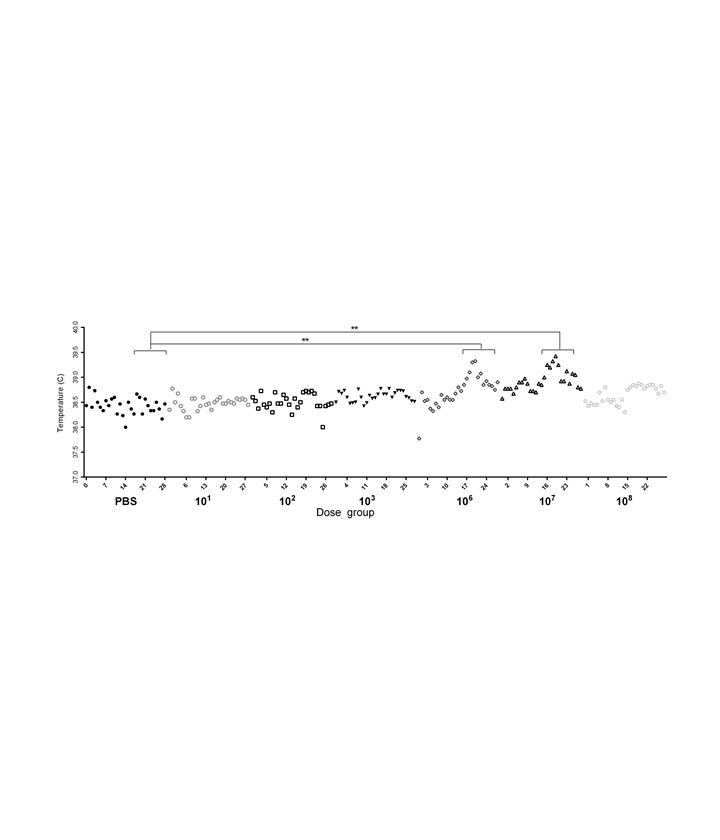

Supplement: S2 Fig — Comparison of body temperature differences between uninfected controls (PBS) and dose groups. Statistical significance by ANOVA followed by Dunnett’s multiple-comparisons. The mean daily temperature was compared between the uninfected controls and the dose groups. Two asterisks, P <0.01. (TIF) [file pone.0212457.s003.tif]

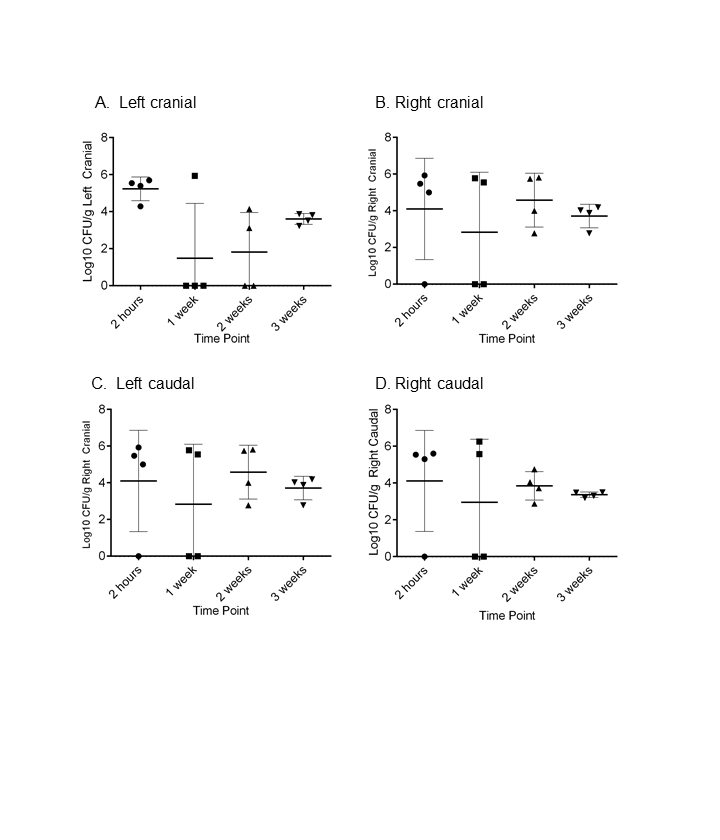

Supplement: S3 Fig — The distribution of aerosolized B. melitensis 16M in the lung lobes of guinea pigs inoculated with 1x10^7^ CFU/50 μl was evaluated at 2-hours and 1,2, and 3-weeks post-inoculation. The lung was divided into four regions defined as left cranial (A) and left caudal (C) and right cranial (B) and right caudal (D), and tissue colonization was determined by region. The horizontal bar is the mean per group with standard deviation. (TIF) [file pone.0212457.s004.tif]

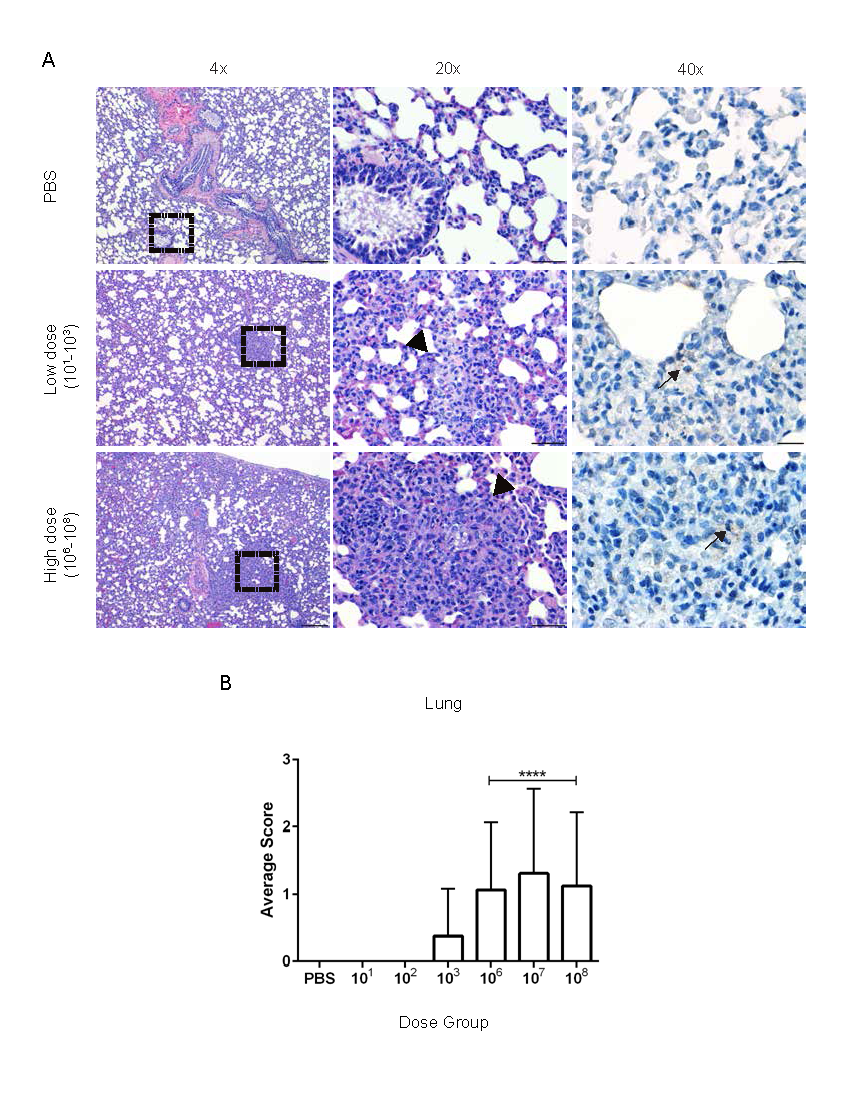

Supplement: S4 Fig — (A) Representative images of histopathology and immunohistochemistry of the lung following intratracheal inoculation with PBS (top), B. melitensis 16M at low dose (middle), high dose (bottom) at 30-days post-inoculation. (B) Sections were scored for severity from 1–4 (S1 Table) based neutrophilic inflammation, number and size of microgranulomas and necrosis, and bronchoalveolar hyperplasia. The black dashed box in the left panel indicates the section highlighted for higher magnification in the middle and right panels. Foci of histiocytic inflammation were seen in the low and high dose groups (arrowheads), but the lesions were larger in the high dose group. Brucella antigen was detected within alveolar macrophages in areas of inflammation by IHC (arrows). Magnification 4x (left, H&E, bar = 200 μm), 20x (middle, H&E, bar = 50 μm), 40x (right, Anti-Brucella IHC, bar = 20 μm). (TIFF) [file pone.0212457.s005.tiff]
